# Improving reuse during the development process for web systems

**DOI:** 10.1038/s41598-024-74643-7

**Published:** 2024-10-14

**Authors:** Ahmed M. El-Halawany, Hamdy K. Elminir, Hazem El-Bakry

**Affiliations:** 1 Faculty of Computers and Information, Mansoura University, Mansoura, 35516 Egypt; 2 Faculty of Engineering, Kafr Elshiekh University, 33735 Kafr Elshiekh, Kafr El Sheikh, Egypt

**Keywords:** Web system reuse, Systematic Approach for web system reuse, Improve web system development, Software, Information technology

## Abstract

Software reuse has emerged as a crucial practice in the software industry, offering significant benefits in time-to-market, and resources management. This is particularly pertinent in web systems development, where the integration of diverse technologies and the varied backgrounds of technical teams pose substantial challenges. The rapid expansion of web systems underscores the urgent need to adopt best practices and methodologies for web system reuse to streamline the development process, reducing effort, cost, and time. This paper aims to identify the key challenges of web system reuse in the context of small and medium-sized software companies in Egypt and Saudi Arabia. Using qualitative research methods, including interviews, focus groups, and participant observations, an empirical study was conducted to examine current reuse practices and understand the root causes of common challenges. Based on the results of the empirical study, a systematic approach was developed to enhance web system reuse during the development process in the context of small and medium-sized software companies in Egypt and Saudi Arabia. The proposed approach addresses critical gaps in current practices, offering practical guidelines to improve efficiency, reduce development time, and enhance overall software quality. This research contributes to the broader discourse on software reuse by providing context-specific insights and adaptable solutions that are relevant to similar markets worldwide.

## Introduction

Software reuse is the process of utilizing predefined software components, codes, documents, and sometimes parts of existing systems to build new software^1^. This practice significantly improves software productivity and quality by reducing development time and costs and by minimizing risks through the use of proven, tested components^2^. However, software reuse is a double-edged sword: while reusing tested components can enhance product quality and reduce development time, indiscriminate reuse can lead to extended development timelines and increased maintenance due to outdated legacy codes^3^.

Various software reuse approaches, such as software product lines, code generation, and Content Management Systems (CMS), have been adapted to meet the diversity of requirements for different industrial situations^3–10^. Although there is significant growth in the demand for both web and mobile applications, web applications remain critically important because many mobile applications rely on web application programming interfaces (web APIs) as backend services^11,12^. The high demand for web applications has prompted software companies to adopt the most suitable reuse approach to meet the rapid growth in the market^2,13^. Web system reuse is still an emerging discipline and a new challenge because the development teams come from different technical backgrounds and utilize a variety of technologies, which can widen the gap in collaborations and increase the complexity of reuse^14–16^.

Few studies have been conducted empirically in real environments to identify the challenges of web system reuse inside software companies^13,15,17–19^. Addressing these challenges requires a comprehensive empirical study of current practices and a deep understanding of the reuse process from various perspectives.

This paper presents an empirical study focused on the challenges of web system reuse during the development process within small and medium-sized software companies in Egypt and Saudi Arabia. Chosen for their exponential market growth and the broader impact of the Egyptian software industry in the Gulf region, these markets present unique opportunities for enhancing software reuse practices^18,20–23^. Through extensive observation of current reuse practices across different development phases, we have identified the main challenges and analyzed their root causes. The analysis of data revealed several key challenges including increased the time and cost to modify a reusable asset, duplicated efforts to build, update, or fix reusable components, and a lack of awareness among technical teams about available reusable components. Based on our findings, a systematic approach was proposed to improve reuse practices during the development process.

This research aims to enhance competitive advantages of Egyptian and Saudi software companies by improving productivity and optimizing project budgets, thereby delivering high-quality, professional web systems. This objective can be achieved by presenting a comprehensive study based on real-world experiences to highlight the challenges of web system reuse and proposing an approach to tackle these challenges.

This paper is structured as follows: Sect. 2 reviews related work, Sect. 3 describes our research methodology, Sect. 4 discusses the results, Sect. 5 outlines our systematic approach for improving reuse, Sect. 6 examines threats to validity, and Sect. 7 concludes the study.

## Related work

The landscape of software reuse is rich with studies addressing challenges across both technical and non-technical reuse domains, including the adaptation of methods and techniques across various industrial settings^24–30^. Despite the breadth of research, many of these studies have not systematically integrated software reuse into their development processes, often overlooking the strategic planning of reuse and its integration into the lifecycle of software development.

Kevin D. Wentzel^31^highlighted a significant barrier to software reuse—cost—with findings suggesting that making a component reusable can add up to 60% in additional costs, deterring many in the industry from integrating reuse practices into their regular workflows^28,32^. Furthermore, the lack of formal documentation for reusable assets, such as Business Requirement Specifications (BRS) and software designs, exacerbates maintenance challenges, leading to increased costs and efforts^33^.

Recent practices among most software developers have shown that they combine components that were not designed to be used together to build a new system^34^. This approach lacks systematic implementation, where developers continually evolve such components to build a new system without sufficient technical experience to select or combine such components effectively.This result is similar to the findings of Jalender et al^19^., which presented the reasons for failure in the product line approach, and these results are consistent with our study as well.

Additionally, Jalender et al^19^. and Younoussi, Siham, et al.18 showed that the software industry has not evolved the reuse activities as a part of the development process and has not afforded the cost of building reusable assets. We examined these results practically during our study and obtained the same results in Egyptian and Saudi software industries.

Younoussi, Siham, et al^18^. investigated the challenges of software reuse practically in the Moroccan software industry by using a descriptive survey as a research method to collect the data from the participants. Their results showed that 82% of the organizations were interested in fully or partially reuse, especially IT service providers’ organizations, but 80%of the organizations lacked guidelines and did not apply the correct practices.

One of the most common challenges for software reuse is that no one in the organization is responsible for promoting reuse, and the modifications or updates in the reusable assets are related to human attitudes and motivation^17,34^. This challenge is also confirmed by Dubinsky, Yael, et al^17^. when they investigated practically the cloning culture in the context of product line reuse methodology. This challenge empirically was investigated during this study and the results showed that the responsibility of reuse in the organizations is missing and anyone can reuse any assets, whether they exist in the organization or on the internet, without any governance.

Furthermore, our literature review highlights a significant gap in empirical research specifically addressing web system reuse challenges in the Middle Eastern context, particularly within the dynamic markets of Egypt and Saudi Arabia. This lack of focused empirical study underscores the originality and necessity of our research. Our study aims to fill this gap by providing detailed empirical insights into the challenges faced by developers in these regions, thereby contributing to a more comprehensive understanding of software reuse practices in these rapidly evolving markets. Through rigorous data collection and analysis, we will explore how regional factors, such as market demands, technological advancements, and local development practices, influence software reuse approaches. This approach not only addresses the highlighted gaps but also enriches the global discourse on software development practices in less-documented markets.

## Research methodology

This study utilized a qualitative research methodology within the framework of an empirical study, as it allows for close examination of practices in real environments^35^. Unlike previous studies, this study bridges the gap between theoretical research and practical implementation by using direct observations and involving direct participation in the development process in real-world scenarios. This approach not only enabled deep engagement with current practices and challenges in web system reuse but also provided new insights into effective strategies for enhancing web system reuse for small and medium-sized companies. The novelty of this work lies in its application of Grounded Theory to systematically analyze and address the specific challenges faced by small and medium-sized software companies in Egypt and Saudi Arabia. Dissimilar to large companies that follow best standard practices and rarely encounter issues in their processes, small and medium-sized companies face unique challenges.

Grounded theory was used as our qualitative data analysis methodology to understand the present state, identify major challenges, and determine their causes. This involved coding the data, identifying categories, and developing themes that emerged from the data^36^. All methods in this study were conducted in strict accordance with relevant guidelines and regulations. The experimental protocols were rigorously reviewed and approved by the ethical committee of the Faculty of Computers and Information at Mansoura University, Egypt. The committee ensures that all research activities comply with the highest ethical standards and regulatory requirements. Informed consent was obtained from all participants and/or their legal guardians before their inclusion in the study. Standardized procedures were followed, and pilot testing was conducted to refine our methodology.

### Data collection

We adopted interviews, focus groups, and observation as qualitative data collection methods to gather information about current practices. Subsequently, we used grounded theory as our qualitative data analysis methodology to systematically collect and analyze the data to construct theories grounded in real-world observations, utilizing ATLAS.ti, a specialized software tool for qualitative data collection and analysis^37^.

### Data analysis

#### Open coding

In the initial stage of data analysis, open coding was used to break down the qualitative data into discrete parts. Codes were assigned to elements such as project size and different technical roles for the company stuff, the available reusable assets and its types, and the development phases followed in the companies.

#### Axial coding

We related the codes which result from the open coding then identified relationships between different codes and grouped them into categories to organize the data and understand the underlying patterns.

#### Selective coding

The core categories are identified then related them to each other in order to develop a cohesive narrative explaining on how various factors influenced web system reuse practices.

#### Validation

To ensure the robustness of our findings, we discussed our emerging themes and categories with the participants to validate our interpretations. This step ensured that our findings accurately reflected the participants’ experiences and addressed their main pains.

#### Integration

Finally, we integrated the refined categories to develop a cohesive narrative explaining how various factors influenced web system reuse practices. Our grounded theory provided a comprehensive understanding of the factors affecting web system reuse, highlighting key challenges and offering insights into best practices for improving reuse effectiveness. Accordingly, we have developed a systematic approach to enhance web system reuse. The proposed approach designed and continuously improved during our participant observations to validate its feasibility and effectiveness in real environment scenarios.

### Limitations and mitigations

While this study provides valuable insights, it is crucial to acknowledge potential biases and several limitations that may influence the findings and to take steps to mitigate them. Below, we discuss the biases and limitations of our study and the strategies we employed to mitigate them:

- **1. Subjectivity and bias**.

#### Limitation

Qualitative research relies heavily on subjective interpretations of data, which can introduce researcher bias or participants’ bias.

**Mitigation strategies**:

- **Triangulation**: Used multiple data sources, including interviews, focus groups, and participant observations, to validate findings.
- **Anonymity and confidentiality**: Participants were assured that their responses would be anonymous and confidential, and would not be linked to their identity or specific company.
- **Peer review**: Engaged external experts in the field of software development to review our data, coding schemes, and findings. This added an additional layer of scrutiny and helped ensure the robustness of our findings.
- **Reflexivity**: Maintained a reflexive approach, documenting and reflecting on our own potential biases throughout the research process.

- **2. Sample size and generalizability**.

#### Limitation

The participant observation was conducted with a sample of 20 companies in Egypt and Saudi Arabia, which may not be representative of all small and medium-sized enterprises globally.

**Mitigation strategies**:

- **Diverse sample selection**: Selected companies based on three primary criteria to capture a broad range of experiences and practices and from various industries and regions within Egypt and Saudi Arabia to ensure a diverse and meaningful sample.
- **Future research**: Suggested that future research include larger, more diverse samples to enhance generalizability.

- **3. Technical awkwardness**.

#### Limitation

Discussing challenges in front of leaders and managers during the focus group sessions presented awkwardness.

#### Mitigation strategies

This limitation was mitigated by dividing stakeholders into two groups based on their roles (management groups and technical groups).

- **4. Observation duration**.

#### Limitation

The duration of participant observation varied from three months to one year, which may not be sufficient to capture all the long-term impacts of reuse practices.

#### Mitigation strategies

This limitation was mitigated by conducting follow-up interviews and maintaining contact with the companies to gather additional data and insights over a longer period. Future studies could extend the observation period to more comprehensively capture long-term effects.

Our initial step targeted the top hundred software companies, that heavily involved in web development, which providing services to organizations, institutions, universities, and government entities in Egypt and Saudi Arabia.

These companies were selected based on three primary criteria:

1. **Market influence**: The market influence was assessed for these companies based on their market share and reputation within the industry. This was determined through publicly available market reports and rankings within industry publications, ensuring that our study focused on key players in the market.
2. **Project volume and product delivery**: The number of projects and the types of products delivered by these companies played a crucial role in their selection. The historical data was analyzed for their completed projects and ongoing engagements, provided insights into their operational scale and the complexity of systems they handle.
3. **Technical workforce size**: The number of technical staff employed by the company indicated their capacity for undertaking substantial software development projects. Companies were segmented into small (less than 50 employees), medium (51 to 150 employees), and large (more than 150 employees) categories based on their technical staff. This categorization helped in understanding the different challenges faced by companies of varying sizes.

The aims and benefits of our study were communicated through personalized emails, explaining how participation could help them identify and tackle the root causes of their challenges, thereby enhancing their competitive edge in the market. The response rates were indicative of the interest and relevance of our study, with small companies showing a 90.4% response rate, medium-sized companies 92.5%, and large companies 50%, culminating in an overall response rate of 84% as shown in Table 1. These rates are consistent with software engineering research standards and comparable to other similar studies conducted in the region^38,39^.

**Table 1:** The number of emailed companies, number of responses, and response rate.

Companies size	Number of emailed companies	Number of response companies	Response rate
Small (< 50)	42	38	90.4%
Medium (51–150)	40	37	92.5%
Large (> 150)	18	9	50%
	100	84	84%

Informed consent was obtained from all participating individuals and their legal guardians to participate with us in this study. All methods were carried out in accordance with relevant guidelines and regulations. Additionally, all experimental protocols were approved by the ethical committee in Faculty of computers and information at Mansoura University, Egypt.

Exploratory interviews were conducted with 354 different technologists from companies which agreed to collaborate with us. The interviews were conducted remotely through video conference using Microsoft Teams, Skype^40^, Zoom^41^or Webex^42^. The maximum duration for the interviews was 30 min. The main purpose of exploratory interviews was focused on how far web system reuse methodologies and practices are applied, identifying at a high level the main challenges of web systems reuse in real environments. During the first round of interviews, the definitions for various terms used during the study were consolidated to set the expectations and remove any conflicts in terminology among the participants because of the different technical backgrounds and cultures for the stakeholders involved from various companies. Therefore, the most commonly used terminology during the study were described and agreed as follows:

- **Satisfaction level of the current practices**.

The satisfaction level of the current reuse practices was addressed into three levels: full satisfied, partially satisfied, and not satisfied. Fully satisfied indicated that the stakeholder is satisfied with the current practices and unwilling to improve them. Partially satisfied indicated that the stakeholder is satisfied and has concerns and seeking improvements for the current practices. Not satisfied indicated that the stakeholder is not satisfied at all with current practices and requests improvements.

- **Company size**.

The company size was defined based on the number of technical employees such as analyst, development managers, technical team leads, project managers, software developers, and quality controllers. The companies with less than 50 employees considered as small-sized companies; those with between 51 and 150 employees were considered medium-sized companies, while those with more than 150 employees were considered large-sized companies.

- **Project size**.

The project size was defined based on the project duration. This duration started from the analysis phase and finished with the testing of the whole project. Projects requiring a period less than five months are considered small-sized projects, between five months and one year as medium-sized projects, and more than one year are considered large-sized projects.

- **Development phases**.

Regardless of the development methodology (waterfall, scrum, agile or spiral) the participants followed inside their companies, agreed with them on four main traditional development phases (analysis, design, implementation, and testing phase).

- **Reusable assets**.

The reusable assets are any documents, pieces of codes, software component, or a part of a web system which can be reused in multiple projects with specific conditions.

According to the data collected during exploratory interviews, a list of open-ended questions was developed to conduct semi-structured interviews, such as:

- Q1: Can you list the existing reusable components, and give a short description of them?
- Q2: How do you integrate an existing component with a new web system?
- Q3: What is the impact of using a reusable component on development time, cost, and productivity?
- Q4: What types of software assets reused? Do they reuse documents, test cases, or only reuse codes?
- Q5: Is there a standard process they follow to build a reusable component, if yes describe it, please!

The purposes of semi-structured interviews depended on the respondents’ professional roles and their objectives inside the company. For example:

1. CEOs and software development managers to discover the impact of web systems reuse on productivity, quality and delivery commitments.
2. Project Managers to explore the impact of web systems reuse on projects plan, quality, modification efforts, and maintenance costs.
3. Software development managers and technical team leaders to understand how they determined which components should be reused and how to reuse them.
4. Software team leaders to identify the software reuse methodology they use and examine the results of applying it in the development process.
5. Software developers to highlight the impact of applying software reuse on their productivity, software quality, and modification and maintenance time.
6. Software quality controller to check the results of applying software reuse to software quality.

**Fig. 1:**
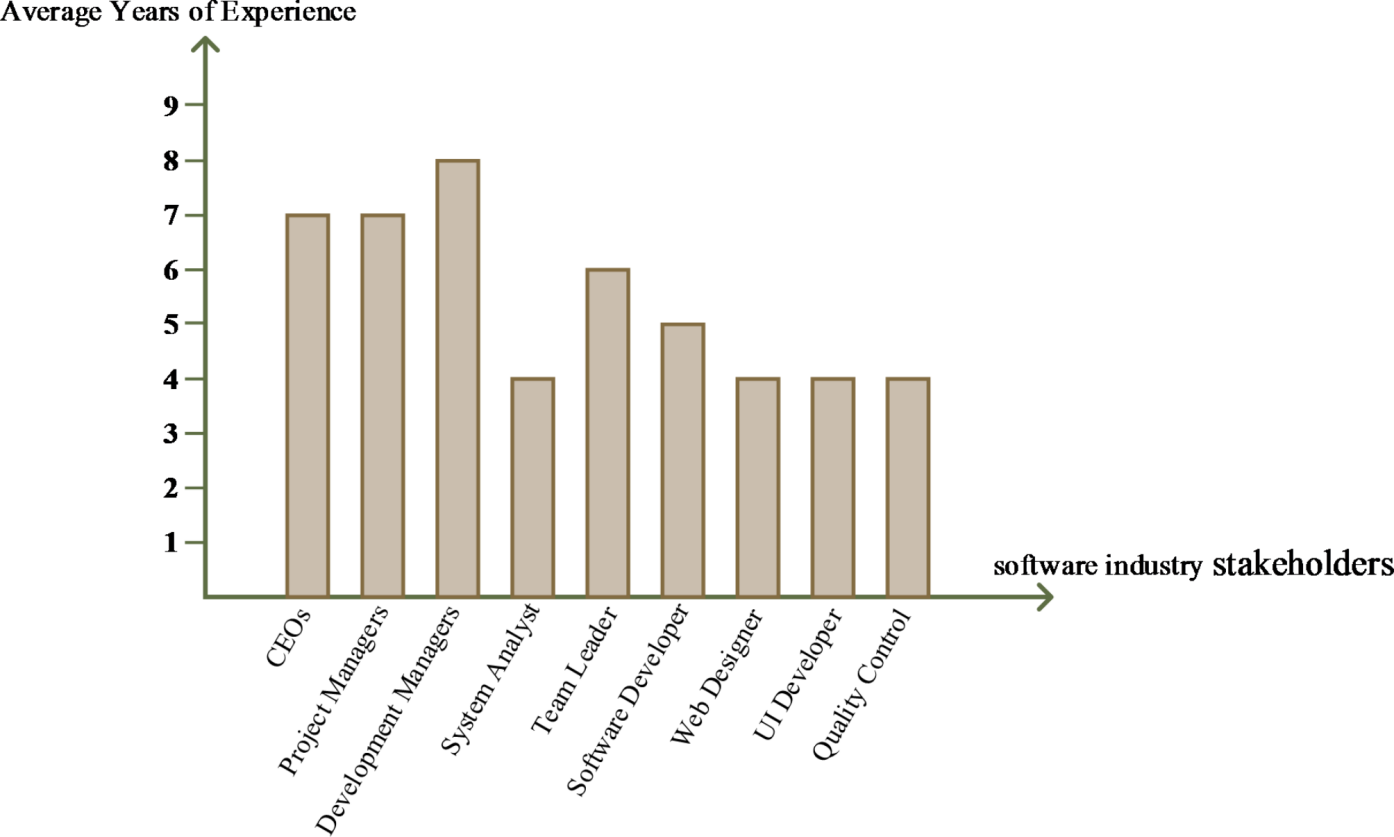
The average years of experience for interviewees categorised by the roles.

Therefore, the questions of semi-structured interviews were categorized based on the interviewee’s role since the interviews followed more of a conversational flow^38^. The average years of experience for interviewees categorized by the roles shown in Fig. 1.

Table 2 shows number of the exploratory, and semi-structured interviews have conducted in the various software industry stakeholders in Egypt and Saudi Arabia.

**Table 2:** Number of conducted interviews with various stakeholders.

Respondents’ role	Exploratory interviews	Semi-Structured interviews	Total number of interviews
Chief executive officer (CEOs)	33	28	61
Project manager	35	45	80
Development manager	40	50	90
System analyst	42	52	94
Software team leader	45	63	108
Senior software engineer	54	101	155
Web designer	23	35	58
UI developer	37	56	93
Quality controller	45	65	110
	**354**	**495**	**849**

Based on the data collected during interviews, we decided to conduct focus groups with different stakeholders in order to have deep understanding of the current web system reuse practices from different perspectives, either within the same company or externally. A categorized contact list was created containing stakeholders who contributed to the interviews. This list helped to organize and conduct focus groups. Stakeholders were divided into two groups based on their roles:

- The management group: This includes CEOs, Development Managers (DM), Project Managers (PM), System Analysts (SA), Team Leaders (TL).
- The technical group: This includes Development Managers (DM), System Analysts (SA), Team Leaders (TL), Software Developers (SD), Web Designer (WD), User Interface Developer (UID), and Quality Controllers (QC).

The focus groups were divided into two types:

- Homogeneous focus groups: These involve stakeholders from the same company within their actual environments.
- Heterogeneous focus groups: These involve stakeholders from different companies.

A list of steps was identified to follow during our study in order to have deep understanding of the reasons for the current challenges as follows:

1. Define which role reported the challenge.
2. Identify the causes of challenge and link them with practices.
3. For each practice, specify the below points:
  - in which phase of the development process this practice occurred.
  - which role performed this practice.
  - Consolidate the reason for this practice during the conducted interviews with stakeholders who performed this practice.
4. Conducted both homogeneous and heterogeneous focus groups with stakeholders who performed and reported these practices to validate our findings during the study.

Table 3 shows the number of various types of focus groups conducted. The following steps were taken to prepare each focus group before it was held:

1. Define the main objectives of the focus group.
2. List the main topics, and sometimes open-ended questions.
3. Determine the required roles that must attend the focus group, and always keep the total number of software stakeholders between five and a maximum of ten participants.
4. Determine the expected duration, proposed location (if the focus group will be conducted in one place), or the proposed application (Skype or zoom) that will be used if it will be conducted remotely.
5. Agree with the selected attendees expected time and topics that will be discussed in the meeting.
6. Send an email to all attendees to confirm the meeting with time, location, and topics.

**Table 3:** Number of various types of focus groups conducted.

Homogeneous/ Heterogeneous		Management group	Technical group	Total number
Homogeneous		23	46	69
Heterogeneous	In one spot	17	50	67
	Remotely	28	63	91
		**68**	**159**	**227**

According to the analysis results of the data collected during focus groups and interviews, we recognized the importance of conducting participant observation to validate the results of the analysis and ensure that challenges and practices collected during interviews and focus groups are consistent with the real environment^43^. We agreed with seven medium-sized companies and thirteen small size companies to conduct participant observation to observe and contribute within the development process, starting from the analysis phase and documenting the requirements passing through the development phase, designing web systems layouts, editing them as HTML, actual implementation for coding system functionality, and finally delivering the web system to the customer. During the observation, the proposed approach was developed, continuously improved, and rigorously tested for its feasibility and effectiveness in real web projects. These results demonstrated the practicality and effectiveness of our proposal in real-world scenarios, providing evidence of its potential benefits for improving web system reuse practices. We contributed to the full development process for 13 web systems, and partially for 32 web systems. We participated in various types of the web systems such as electronic services for government sectors (E-Services), Enterprise Resource Planning (ERP), archiving web systems, Enterprise Project Management Systems (EPM), and Content Management systems (CMS) for small organizations. Table 4 shows the number and types of the observed web systems. The participant observation took approximately three months for some systems and extended to more than one year for others. This period was calculated starting from the development phase after the customer approved at least one requirement document until the customer received the first release.

**Table 4:** Number of observed web systems and its types.

Web system type	Fully observed	Partially observed	Total number
E-Services for government sectors	2	6	8
ERP web-based system	1	3	4
Archiving web-based system	1	4	5
Management system for organization	2	6	8
EPM	1	4	5
CMS	6	8	14
Online shopping	0	1	1
	**13**	**32**	**43**

## Results and discussion

The primary objectives of this study were to identify the challenges faced by small and medium-sized software companies in web system reuse and to develop a systematic approach for enhancing reuse practices. The key research questions guiding this study were:

1. What are the main challenges in web system reuse?
2. How can a systematic approach improve web system reuse practices in these companies?

In this section, the first research question will be addressed by highlighting comprehensive insights into the current web system reuse practices and challenges across various company sizes, focused on small and medium-size companies. In the next section, the second research question will be addressed by presenting a systematic approach to enhance the web system reuse practices.

The results reveal significant disparities in satisfaction levels across different company sizes, with large companies generally more content with their reuse practices compared to their smaller counterparts. The findings also underscore a common set of challenges that impede effective reuse, highlighting the specific impacts on quality, cost, and developmental stress. Additionally, the data suggest a notable misalignment between project management plans and development execution, which often exacerbates the challenges faced during the reuse processes.

Key Findings to be Discussed:

1. **Satisfaction levels across company sizes**: An analysis of the varying degrees of satisfaction with reuse practices among large, medium, and small-sized companies.
2. **Common challenges in reuse practices**: A detailed examination of the challenges commonly reported across companies, focusing on their implications for operational efficiency and project success.
3. **Impact of poor reuse practices**: Insights into how suboptimal reuse practices affect project quality, increase costs, and place undue pressure on development teams.
4. **Differing stakeholder perspectives**: An exploration of the differing views between management and developers, and the role of project managers in bridging these perspectives to enhance project outcomes.

### Satisfaction levels across company sizes

In the initial phase of the conducted interviews and focus groups, the level of satisfaction with the current reuse practices was measured inside small, medium and large-sized company across all project size. The results of data analysis for the conducted interviews showed that 86% of stakeholders from large companies were completely satisfied with their reuse practices, while the remaining 13% were partially satisfied but had some concerns and were interested in improving their reuse practices during the development process. Only 1% of the participants from large companies were not satisfied with their practices.

In medium-sized companies, 78% of stakeholders were not satisfied with their reuse practices and 14% were partially satisfied. An unexpected finding was addressed in small-sized companies, where a significant percentage (68%) reported satisfaction with their reuse practices. This satisfaction can be attributed to the nature of their work on small or medium web systems, which are delivered in a short time at low cost. These companies effectively utilized Content Management Systems (CMS) like Joomla^44^, web application frameworks like Laravel^45^, or low-code platform like OutSystems^46^.The remaining 32% of stakeholders from the small-sized companies were either not satisfied or partially satisfied with their reuse practices and were interested in improving their practices because they were developing medium and large web systems that contained tailored subsystems and components. Therefore, they sought to deliver these systems with better quality and less time to gain competitive advantages.

**Fig. 2:**
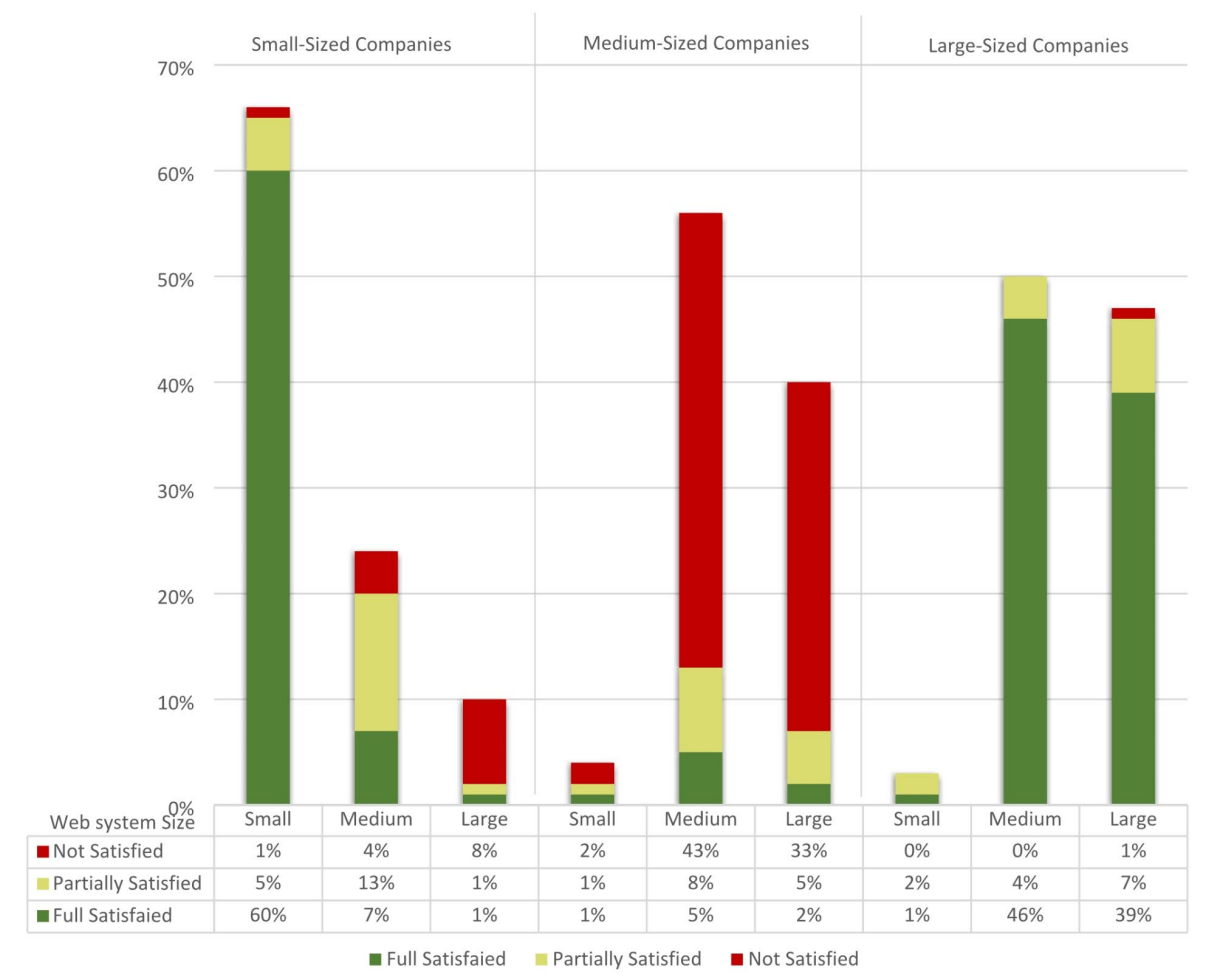
The percentage of stakeholders from various company sizes, and rated their satisfaction across the different sizes of web systems.

Figure 2 in the chart below shows the percentage of stakeholders who contributed to the interviews from various company sizes and rated their satisfaction with their reuse practices across the different sizes of web systems as satisfied, partially satisfied, or not satisfied.

### Common challenges in reuse practices

The results of data analysis for interviews showed that about 92% of stakeholders who seek to improve their reuse practices belong to medium-sized companies, about 32% from small-sized companies, and about 14% from large-sized companies. Consequently, we found that the pain is concentrated in medium and small size companies which deliver medium and large-scale web systems. Therefore, categorized focus groups were conducted with emphasis on various stakeholders in medium and small-size companies.

According to the data analysis results for focus groups, the CEOs and Project Managers (PMs) preferred to build the new web systems based on the existing components, especially when customers request their web system urgently, but they complained about the poor quality and longtime of implementation, especially when required to develop the new requirements for the customer in the future.

Unexpected findings were addressed in the effort estimation process, where 37% of project managers used their experience to estimate effort for the implementation timeframes and committed their plan to the customers without alignments with the development team. A direct question was asked to this group of project managers during the focus groups “Have you committed to a plan with customers without alignment with the development team?”, the answer was very clear “Yes” when asked “why?” they reported that sometimes, to sign a contract with the customer, they need to accept the project regardless of the customer’s restrictions on the deadline, and they will not wait until the development team reviews the project scope and shares their effort estimation. This practice occurs because the project managers assumed that sometimes the project scope is already implemented in previous projects so the development team can reuse and update it according to the new project scope easily.

The development team reported that they always worked under pressure because CEOs and PMs thought software reuse was the magic wand that could develop the various types of web systems with different sizes in less time and at the lowest cost. The big surprise for us was that neither the development manager, technical team leads nor software developers could identify exactly the existing reusable components inside their company. Even for the identified components, the development team was not particularly aware of the main features of the reusable components or how to reuse them in a new web system, and no documentation was available for the reusable components. Furthermore, when technical team leads planned to implement a reusable component, they did not provide detailed design documentation or review codes after implementation. Additionally, most of the software developers used ad-hoc reuse in which they copied a block of code or a component from an existing web system to a new one. The responsibility or ownership of the reusable components were not exists therefore, software developers could reuse the old component indiscriminately and did not pay attention to whether they were reusing the last updated version of the component or not. A repeated case was addressed where, in the same company, two teams working on two different projects each team updated the same reusable component with identical new features in both projects. Based on the feedback from the development team, the percentage of the tested reusable components in a new web system was calculated: it was 17%, and the remaining 83% of the existing reusable components were reused without full or partial testing. UI developers used common frameworks to edit the frontend for a web system, and they used both Cascading Style Sheets (CSS) frameworks like “Bootstrap”^47^or “Materialize”^48^; and JavaScript frameworks like “angular”^49^or “jQuery”^50^. The results showed that 77% of UI developers started editing frontend for a web system from scratch while the remaining 33% of them made their standard reusable library categorized by framework.

### Impact of poor reuse practices

**Fig. 3:**
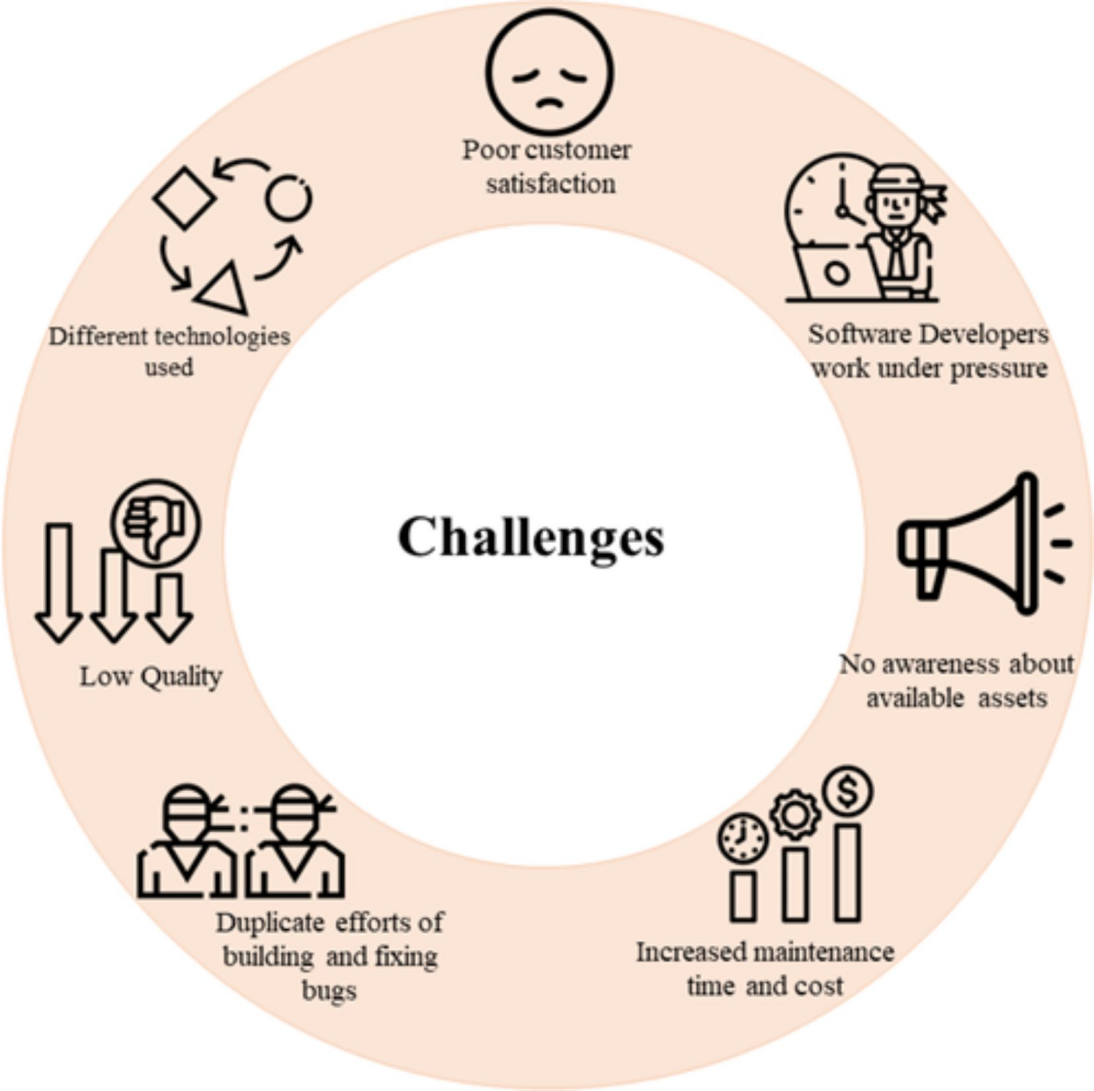
List of common complaints, causes, and wrong practices.

Participant observations inside 20 software company was conducted to verify the results of data analysis for interviews and focus groups, in addition to gaining a deep understanding of the current practices and identifying the challenges of web system reuse. The main objective of the first term of the observations was to compare the results of interviews and focus groups with the current state of the observed companies. The results of the observation were identical to the results of the interviews and focus groups. Accordingly, the results of data analysis for interviews, focus groups and participant observations presented in Fig. 3. It illustrated the commonly shared challenges of web system reuse inside the medium and small-sized companies in Egypt and Saudi Arabia.

In Table 5, we listed the commonly shared challenges and set an ID for each one, categorized by the roles who reported these challenges and the percentages of stakeholders who report this challenge from the same role. The results showed that the top-level management (CEOs, project managers) were frustrated by poor customer satisfaction and increased maintenance time and cost due to the low quality of the delivered web systems while development managers did not perceive these facts. Awareness about the available reusable components inside the company was lacking; moreover, software developers were the stakeholders who suffered the most, working under pressure because they exerted more effort to fix reusable components’ bugs or built new components based on existing ones without technical documentation. The system analysts did not share any challenges related to reusing the existing components or documents. Most of their challenges were related to gathering requirements and getting approval from customers on the requirements, so we have not mentioned these challenges as they are not related to the scope of our research.

**Table 5:** Common challenges of web system reuse, who role reported, and the percentages who voted to challenge.

ID	Challenges	Reported percentages by role							
		CEOs	PM	DM	SA	TL	SD	UID	QC
C1	Poor customer satisfaction	78%	83%	40%	☒	☒	☒	☒	☒
C2	Increase the cost of modification in a reusable component	88%	81%	47%	☒	☒	☒	☒	☒
C3	Increase the time of modification in a reusable component	80%	78%	43%	☒	40%	☒	☒	☒
C4	Duplicate efforts to build or update a reusable component	☒	66%	53%	☒	76%	86%	75%	☒
C5	Duplicate the efforts of fixing bugs for a reusable component	☒	76%	57%	☒	43%	80%	35%	86%
C6	Technical Stakeholders always work under pressure	☒	☒	☒	☒	56%	81%	58%	59%
C7	Low software quality	61%	70%	42%	☒	☒	☒	☒	62%
C8	Different technologies used in the same component	☒	☒	77%	☒	80%	79%	48%	☒
C9	No awareness about the available reusable components.	☒	☒	57%	☒	72%	79%	74%	☒

We summarized in Table 6, the list of current practices for reusing a web system which caused the above list of challenges.

**Table 6:** The list of current practices for reusing a web system which caused the above list of challenges.

ID	Practices	C1	C2	C3	C4	C5	C6	C7	C8	C9
P1	Reusing existing component while it is not yet ready for reuse because it still contains bugs or still underdevelopment.	☑	☑	☑	☑	☑	☒	☑	☒	☒
P2	Moving web systems which contains reusable components from the development phase to the testing phase without regression testing.	☑	☒	☑	☒	☑	☒	☑	☒	☒
P3	Building reusable components without technical documentation.	☒	☑	☑	☑	☑	☑	☑	☑	☑
P4	Building or updating the reusable components without internal coordination with the development team.	☒	☒	☒	☑	☒	☑	☒	☑	☑
P5	Project managers did not coordinate with the development team to handle the customer restrictions on the deadline.	☒	☒	☒	☒	☒	☑	☒	☒	☒
P6	The tasks of the analysis team are limited to the creation of BRDs and not support in the development process.	☒	☒	☒	☑	☒	☑	☒	☒	☑
P7	Various developers fix bugs in multiple projects related to the same shared component.	☒	☑	☑	☒	☑	☑	☑	☒	☒

### Differing stakeholder perspectives

The results showed that software developers were reusing existing components even though they were not yet ready for reuse because they had not been tested or were still under development. This practice resulted in poor quality, duplicated the efforts of updating or fixing bugs, and increased the maintenance cost and time accordingly. Sometimes the development team moves the web system which contains reusable components, from the development phase to the testing phase without validating whether the plugged-in reusable components are working as expected or not. This practice leads to poor quality and additional time spent to fix the resulting bugs. Building reusable components without technical documentation is one of the practices that has a huge impact because it leads to low software quality, duplicate efforts to update or fix bugs, and increased the time and cost accordingly. The development team works on reusable components either by fixing bugs, updating new features, or creating new ones without internal coordination with other team members, resulting in multiple developers working on the same component in various projects. This practice led to duplicate the efforts by the development team and made them always work under stress. Another practice that constantly stressed the development teams was that project managers set a tight plan for the technical implementation without alignment with the development team. In order to mitigate this situation, the development team started a resource allocation process to add more resources. This process took an average of two weeks or more, during which the development team worked under stress. The analysis team tasks are limited only to providing BRDs and getting approval; they did not engage in any future activities. This practice did not support the development team in extracting the reusable components from the exiting web system and led them to invest more time in creating components as a reusable asset, even though these components might not be reused in any future web systems or might already exists and have been implemented earlier by different teams internally.

However, it is important note that this study presents anonymous individual results since we were trying to obtain access to some sensitive data about individuals and companies.

## A systematic approach for improving web system reuse

**Fig. 4:**
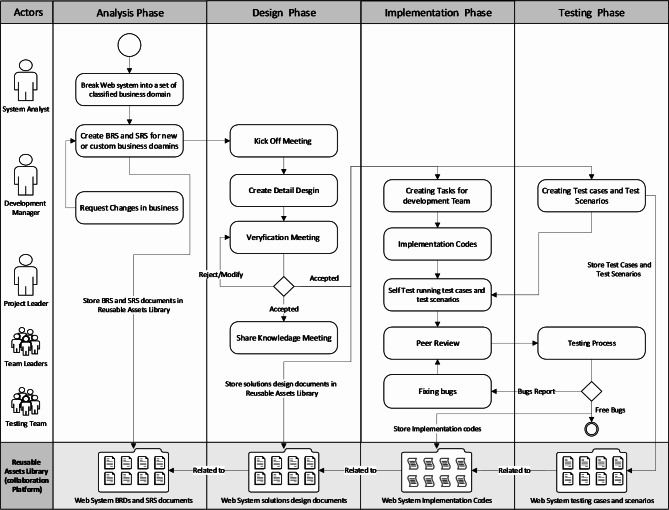
Overview of the systematic approach for improving web system reuse.

In this section, the second research question will be addressed based on insights garnered from our study. Including participant observations of approximately 45 web systems across 20 software companies, a systematic approach was developed to enhance web system reuse. This approach is designed to seamlessly integrate into the existing development workflows, irrespective of the development methodology employed—be it Agile, Scrum, Waterfall, Lean, or DevOps. This approach organizes activities across the four main development phases: analysis, design, implementation, and testing. These phases are outlined in a workflow process depicted in Fig. 4.

**Fig. 5:**
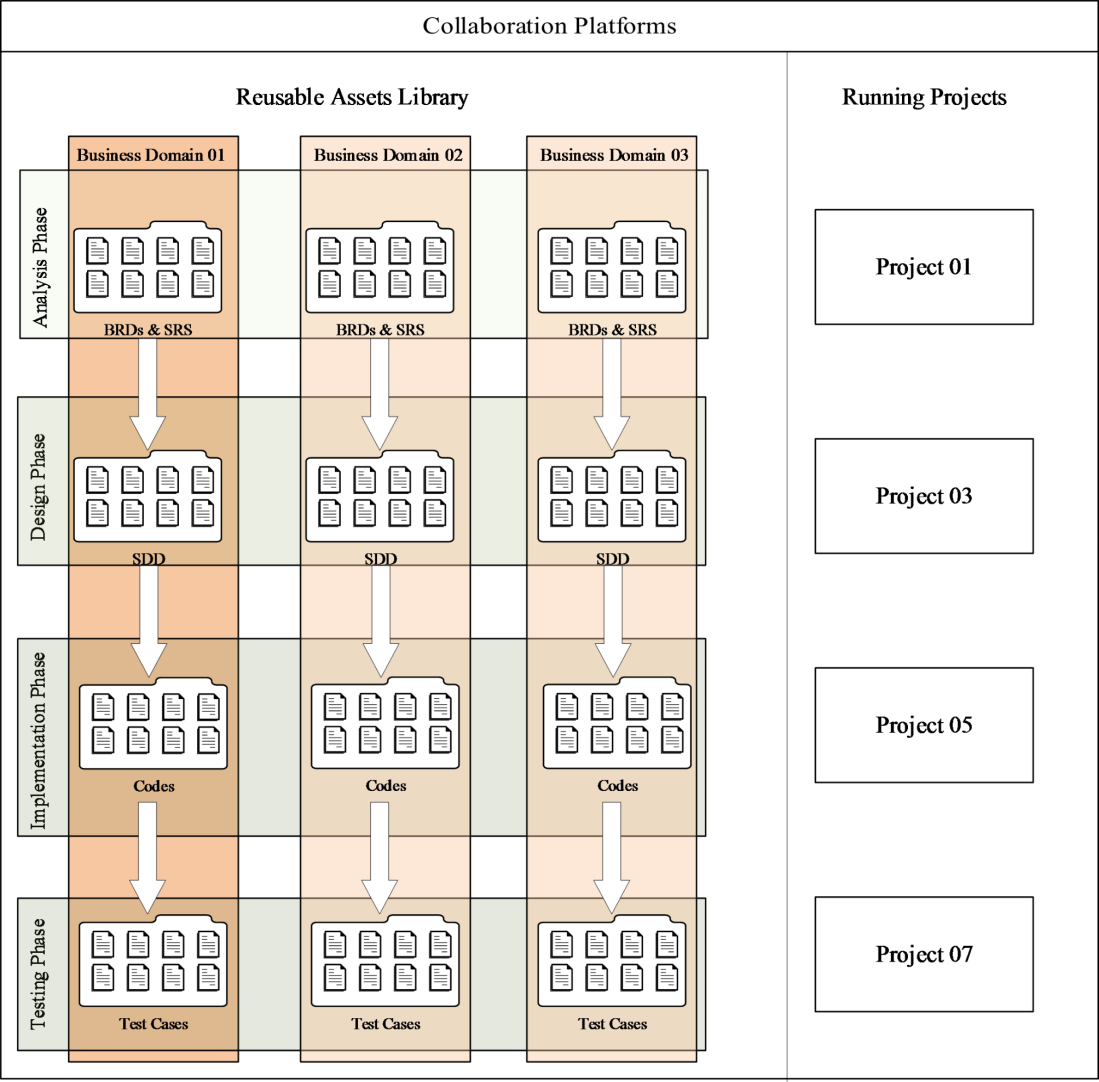
Collaboration platform for improving web system reuse.

This approach proposes using a collaboration platform like Microsoft Team Foundation Server (TFS), AZURE DevOps^51^, JIRA integrated with Bitbucket^52^, or any similar platforms to maintain and organize all reusable assets in one repository. The collaboration platform should be divided logically into two areas: the first one is the reusable assets library, and the second one is the running project area, which is configured according to the development methodology used by the company or running projects. The reusable assets library area should be categorized by the reusable business domains. Each domain should maintain its BRDs related to its SRS documents, related with its solution design documents (SDD), and associated implementation codes along with test cases and test scenarios, as shown in Fig. 5.

### Analysis phase

In the analysis phase, the system analyst plays a pivotal role in deconstructing the new web system into distinct business domains. These domains are categorized based on their reuse potential and requirement specifics:

1. **New business domains**: Require analysis from scratch to understand the requirements and then create Business Requirements Documents (BRD) and related System requirements specifications Documents (SRS).
2. **Existing business domains**: Identical to previously analyzed requirements, with their related BRDs and SRS documents already existing.
3. **Custom business domains**: Similar to previously analyzed requirements but need minor updates for their BRDs and SRS documents to fit the new requirements.

The system analyst also calssifies the new business domains into the following two categories:

1. **Domains for reuse**: Anticipated to be reused and are documented with a high level of abstraction to facilitate future integration.
2. **Domains without reuse intention**: Specific to current projects and not intended for future reuse.

**Fig. 6:**
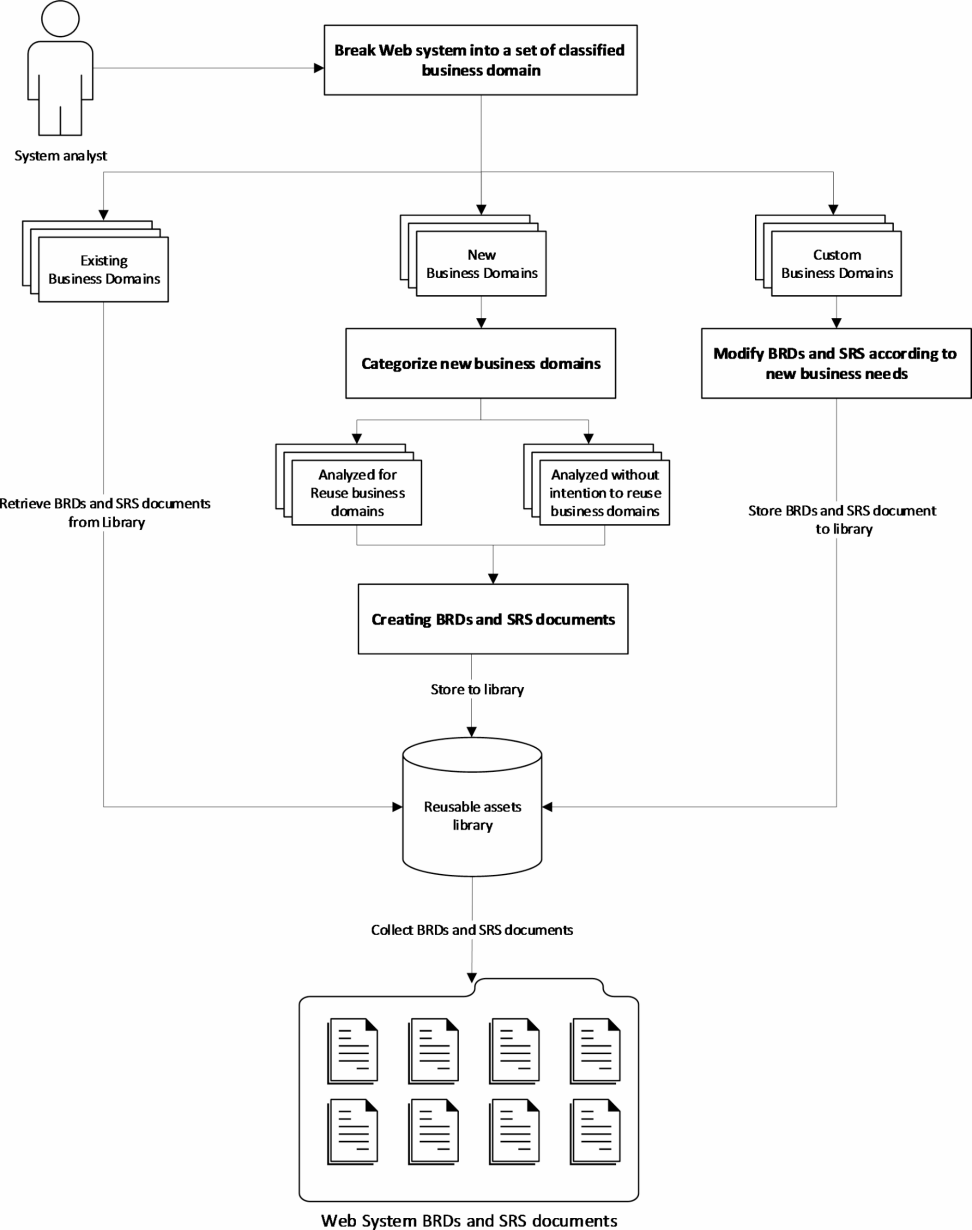
Overview of activities in the analysis phase.

This approach proposes that the system analyst must utilize UML use cases and activity diagrams for detailed documentation for both BRDs and related SRS, stored in the reusable assets library for easy access and update management. This ensures that any adjustments due to customer requests or project adaptations are meticulously tracked, maintaining a version history for all changes. Consequently, in the future, if the system analysts work on a business domain that exists in the reusable assets library, they can reuse the existing BRDs or SRS documents instead of creating a new one from scratch. Figure 6 shows an overview of the activities in the analysis phase.

### Design phase

The design phase begins with a kick-off meeting involving key project stakeholders to align on the project’s scope and the integration of existing business domains into the new project. The software development manager, technical team leaders, testing team, and the system analyst should attend this meeting to discuss and demonstrate the scope of the new project.

The development manager or solutions architect is responsible for dividing these domains into a set of interconnected components related to their SRS documents in addition to creating high-level design documents for the new components. The components are divided into three types as follows:

1. **Developed for reuse**: will be reused in other web systems.
2. **Developed with reuse**: will be built based on existing components.
3. **Developed without reuse intentions**: Not intended for reuse .

The development manager or solutions architect is also responsible for notifying all technical teams about the new reusable components that are either planned to be developed or are under development. Technical team lead for the project is responsible for creating detailed design documents for the new components in the web system based on the high-level design documents. The technical team lead should document detailed design using component diagrams and class diagrams, and if necessary, use state machine diagrams or sequence diagrams. They should also follow the separation of concerns and Model Driven Development (MDD) principles to facilitate reusing these components in different web systems.

**Fig. 7:**
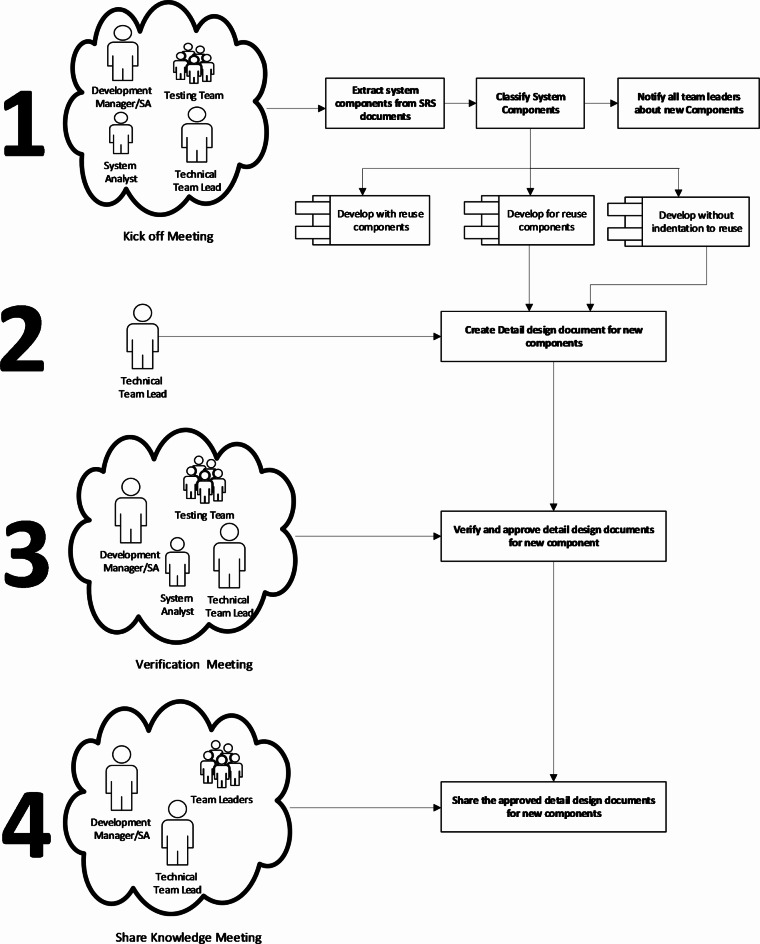
Overview of Activities in the Design Phase.

Once the technical team lead finished creating detailed design documents, the development manager calls for two sequential meetings. Firstly, a verification meeting with the system analyst, technical team leader and testing team to verify and approve the detailed design referring to SRS documents. Secondly, a an awareness meeting with all team leaders in the company to ensure they are aware of the detailed design of components that will be developed for reuse. Figure 7 shows an overview of the activities in the design phase.

The main objectives for the activities during the design phase are to prevent the duplication of efforts for creating existing components, force the development team to reuse proven tested components, and ensure strong alignment among development teams about the available reusable components.

### Implementation and testing phase

The implementation and testing phases should start in parallel to optimize efficiency. In the implementation phase, the technical team lead breaks each new component developed for reuse into a set of small tasks recorded on the collaboration platform and then assigns these tasks to team members to start implementation. At the same time, the testing team creates test cases and test scenarios and then records them in the collaboration platform associated with their BRDs, SRS, and detailed design documents. The testing team should finish recording test scenarios before the technical team finishes the implementation. In order to increase software quality for the new components developed for reused, there are two main activities that must be followed: first, the technical team must review codes and ensure that the necessary tasks are completed. The second activity is applied either when the development team finishes the technical implementations and peer review process for the new component or when the technical team plugs a reusable component into a web system under development. They must run all test scenarios on their codes to ensure their quality before moving to the actual testing phase. The testing team starts the actual testing activities and records all bugs on the collaboration tool, assigning these bugs to the technical team lead to fix them. The technical team lead is responsible for fixing these bugs either by themselves or by another team member and then sending it back to testing. The process is repeated until the testing team accepts and ensures the quality of the software.

For the components developed for reuse, once the development is finished and these components have passed testing activities, the development manager or solutions architect should notify all technical teams and store these new components in the reusable assets library associated with the related SRS, detailed design documents and test scenarios.

If the customer requests changes, the system analyst updates the BRDs and SRS documents and saves them in the collaboration platform as a new version, then start the activities identified in the proposed approach across the four main phases.

### Case study in real world

#### Scenario

Contribution with a Saudi company to build their informative and e-services portal for a government entity in Saudi Arabia.

**Analysis phase**.

The business domains were categorized into three categories:

1. New business domains are categorized into:
  - Domains for Reuse: such as contact us form.
  - Domains without Reuse intention: such as issuing a new licence, submitting objection.
2. Existing business domain: such as news, events, media centre.
3. Custom business domain: such as requesting appointments.

The requirements were documented using UML use case and activity diagrams. Once approved by the client, they were stored in JIRA as Confluence.

**Design phase**.

In this phase, the following steps were followed:

1. **Kick off meeting**: conducted with analyst, technical lead, solution architect, project manager, and testing lead to understand the high-level requirements and project scope. The outcome of kick off meeting is shown in Table 7 below.
2. **High-Level design**: The solution architect created the high-level design documents for new components, stored them in JIRA as Confluence associated with their requirements.
3. **Announcement**: The solution architect sent emails to all technical team leads sharing the high-level design documents for the new reusable components which stored on JIRA.
4. **Detailed design**: The technical team lead created the detailed design documents for the new components and stored them on JIRA as Confluence associated with their high-level design.
5. **Review meeting**: Conducted with project stakeholders to verify and approve the detailed design.
6. **Awareness session**: Held with all technical leads and solutions architect to share with them the new reusable components details stored on JIRA.

**Table 7:** The list of components and its types required for the project.

Business domains	Components		
	Develop for reuse	Develop with reuse	Developed without reuse intentions
Contact us form	■ Contact us form ■ Send notification	X	X
News	X	✓	X
Events	X	✓	X
Media centre	X	✓	X
Request appointment	X	✓	X
Issue a new licence	■ Send Email	X	Issue a new licence
Submit objection	X	X	Submit objection

**Implementation and testing Phase**.

Both phases started in parallel to optimize efficiency as follow:

1. **Task breakdown**: The technical team lead broke down components into tasks associated with detail design, all stored on JIRA.
2. **Test scenarios**: The testing team created the test scenarios for reusable components and stored them on JIRA.
3. **Development**: The technical team members worked on tasks assigned to them on JIRA and push source codes to GitLab which integrated with JIRA.
4. **Peer review**: Once tasks were completed, a peer review process was initiated, where the technical team lead reviewed the code related to each component.
5. **Test execution**: The team lead ran all test scenarios stored on JIRA associated with the reusable component.
6. **Component testing**: The reusable component was moved to the testing team for examination.
7. **Bug tracking**: Bugs related to the component were recorded on JIRA, associated with the source code, detailed design, and requirements.
8. **Bug fixing**: The technical team lead assigned the bugs to a team member to fix the issue then revert it back to the testing team to validate.

The steps from #6 to #8 were repeated until all bugs were fixed and the testing team approved the component

## Threats to validity

In this study, we employed a qualitative research methodology designed to minimize the threats to validity as comprehensively as possible. The potential threats were discussed concerning reliability, construct validity, internal validity, and external validity, and the measures taken to mitigate these concerns.

### Reliability

To ensure the reliability of our findings, three qualitative research methods were used: interviews, focus groups, and participant observation. Approximately 849 interviews and 227 focus groups were conducted with technologists from various technical backgrounds. Additionally, the development processes were observed in real environments within 20 software companies across 43 different web system projects. The consistent results across these diverse methods and settings reinforce the reliability of our conclusions regarding the prevailing practices and challenges in web system reuse within small and medium-sized companies in the Egyptian and Saudi software markets.

### Construct validity

Construct validity was addressed by engaging a wide range of participants, including project managers, technical leads, and testing teams, each providing insights based on their specific roles and experiences. To counteract potential biases and enhance the diversity of perspectives, the focus groups were organized into Management or Technical groups and further into Homogeneous or Heterogeneous groups. This approach allowed us to capture varied perspectives within the same session, enriching our understanding of web system reuse challenges. Furthermore, considering the variance in company sizes and the diverse cultures of participants, terminology was standardized during the preliminary phase of the study to ensure clear and consistent communication throughout the research process.

### Internal validity

Internal validity concerns were systematically addressed by clearly identifying the main challenges associated with web system reuse in small and medium-sized companies. We meticulously documented the practices leading to each identified challenge, ensuring a robust linkage between observed phenomena and our interpretations. This methodological rigor helps substantiate the causative relationships within our findings.

### External validity

While our study initially included participants from various company sizes, the findings indicated that the principal challenges were predominantly present in small and medium-sized companies. Consequently, the participant observations were deliberately focused on these companies, particularly those engaged in medium to large-scale projects, to better understand the context-specific challenges. This focus enhances the generalizability of our results within similar business environments but may limit the applicability of our findings to larger organizations or different geopolitical contexts.

These measures collectively strengthen the validity of our study, providing a solid foundation for the reliability and applicability of our findings. Nonetheless, as with any research, certain limitations remain, and results should be interpreted with consideration of these contextual factors.

## Conclusion

In this paper, an in-depth empirical study was conducted utilizing qualitative research methodologies—including interviews, focus groups, and participant observations—to identify the main challenges of web system reuse across the four main phases of the software development life cycle (analysis, design, implementation, and testing) within small and medium-sized software companies in Egypt and Saudi Arabia. The results reveal significant challenges, notably the ambiguity surrounding the availability and management of reusable assets and the disparate technologies employed during development. These issues have been shown to contribute to decreased software quality, increased development time and costs, and diminished customer satisfaction.

To address these challenges, a systematic approach was developed and to enhance reusability throughout the development process. This approach is designed to improve development productivity and elevate software quality to effectively meet market demands. By implementing this approach, companies can ensure a more efficient reuse of assets, leading to more consistent outcomes and better alignment with customer expectations.

Furthermore, this study highlights the critical need for better-structured and managed reuse processes within the industry, suggesting that the adoption of our proposed approach could serve as a blueprint for companies aiming to refine their software development practices. As the demand for rapid and high-quality software solutions continues to grow, particularly in dynamic markets like Egypt and Saudi Arabia, the insights and methodologies outlined in this paper provide valuable guidance for enhancing competitiveness and operational efficiency in the software development sector.

By systematically addressing the identified challenges and applying the strategies outlined in our approach, companies can not only improve their operational performance but also significantly enhance their market positioning by delivering higher quality products in a more timely and cost-effective manner.

## Future research

Based on the findings of this study, several future research directions and open questions have emerged that warrant further investigation. Future research should investigate the role of emerging technologies, such as artificial intelligence, machine learning, and automated code generation, in enhancing web system reuse practices. These technologies have the potential to significantly improve efficiency and reduce the cognitive load on developers. One key question is: what are the long-term effects of implementing systematic reuse practices on software quality and maintenance costs? Additionally, it is important to include a larger and more diverse sample of small and medium-sized software companies from different geographic regions and industries. This would provide a broader understanding of web system reuse practices and challenges across various contexts.

## Data Availability

The data collected and/or analyzed during this study are not publicly available due to confidentiality agreements with the participating companies. These companies consider the data private and integral to their internal policies. Any exposure of sensitive information or violation of their internal processes could potentially impact their competitive position in the market. Data may be available from the corresponding author, Ahmed M. El-Halawany, upon reasonable request and with permission from the participating companies.
